# Molecular evolution of type 2 porcine reproductive and respiratory syndrome viruses circulating in Vietnam from 2007 to 2015

**DOI:** 10.1186/s12917-016-0885-3

**Published:** 2016-11-17

**Authors:** Hai Quynh Do, Dinh Thau Trinh, Thi Lan Nguyen, Thi Thu Hang Vu, Duc Duong Than, Thi Van Lo, Minjoo Yeom, Daesub Song, SeEun Choe, Dong-Jun An, Van Phan Le

**Affiliations:** 1 Faculty of Veterinary Medicine, Vietnam National University of Agriculture, Hanoi, Vietnam; 2 Research and Development Laboratory, Avac Vietnam Company Limited (AVAC), Hung Yen, Vietnam; 3 College of Pharmacy, Korea University, Sejong, Republic of Korea; 4 Animal and Plant Quarantine Agency, Gyeonggi-do, Gimcheon, Gyeongsangbukdo Republic of Korea

**Keywords:** PRRSV, Vietnam, ORF5, Phylogeny

## Abstract

**Background:**

Porcine respiratory and reproductive syndrome (PRRS) virus is one of the most economically significant pathogens in the Vietnamese swine industry. ORF5, which participates in many functional processes, including virion assembly, entry of the virus into the host cell, and viral adaptation to the host immune response, has been widely used in molecular evolution and phylogeny studies. Knowing of molecular evolution of PRRSV fields strains might contribute to PRRS control in Vietnam.

**Results:**

The results showed that phylogenetic analysis indicated that all strains belonged to sub-lineages 8.7 and 5.1. The nucleotide and amino acid identities between strains were 84.5–100% and 82–100%, respectively. Furthermore, the results revealed differences in nucleotide and amino acid identities between the 2 sub-lineage groups. N-glycosylation prediction identified 7 potential N-glycosylation sites and 11 glycotypes. Analyses of the GP5 sequences, revealed 7 sites under positive selective pressure and 25 under negative selective pressure.

**Conclusions:**

Phylogenetic analysis based on ORF5 sequence indicated the diversity of PRRSV in Vietnam. Furthermore, the variance of N-glycosylation sites and position under selective pressure were demonstrated. This study expands existing knowledge on the genetic diversity and evolution of PRRSV in Vietnam and assists the effective strategies for PRRS vaccine development in Vietnam.

**Electronic supplementary material:**

The online version of this article (doi:10.1186/s12917-016-0885-3) contains supplementary material, which is available to authorized users.

## Background

Porcine reproductive and respiratory syndrome (PRRS) is a major infectious disease affecting pork industries worldwide. Its outbreaks were first reported in the USA and EU in the late 1980s and early 1990s, respectively [4, 5, 42]. The main clinical signs of the disease are respiratory problems in pigs of all ages and reproductive failure in pregnant sows. In Vietnam, PRRS outbreaks have continuously occurred since 2007 [8, 23, 28]. PRRS viruses, the causative agents of the disease, can be divided into two distinct genotypes, type I (EU type) and type II (American type), which present with identical disease symptoms, despite their genetic differences [24]. PRRSV is a mono-partite, linear, positive-sense single stranded RNA virus belonging to the *Arterviridae* family [5]. Its genome of approximately15 kb in size is organized into 10 open reading frames (ORFs) [24, 38]. Two large ORF1a and ORF1b genes encode non-structural proteins that play important roles in viral replication and virulence [13, 18]. The other ORFs encode for structural proteins that are necessary for production of infectious virions [44]. ORF5, which participates in many functional processes, including virion assembly [44], entry of the virus into the host cell [7], and viral adaptation to the host immune response [41], has been widely used in molecular evolution and phylogeny studies [30, 34, 35].

Evolutionary studies indicate that PRRSV diverged long before the first detected outbreaks of the disease. Evolutional analyses based on ORF5, as well as serological evidence, indicated that PRRSV type 2 first appeared around the 1980s [3, 35, 48]. In contrast, type 1 PRRSV originated approximately 100 years ago [30]. Further analysis of the whole PRRSV genome shows that the two types of PRRSV diverged from a common ancestor about 800 years ago [46]. Furthermore, genetic analyses indicate that the evolutionary trends, antigenic characteristics, and genetic diversity of PRRSV in different regions have distinct patterns [6, 11, 17, 32, 36, 40].

Thus far, type 2 PRRSV has been divided into 10 sub-lineages, including 9 old sub-lineages [34] occurring worldwide, and a new sub-lineage, which recently appeared in Thailand [40]. In Vietnam, several studies show that the circulating PRRSV strains belong to a highly pathogenic (HP) variant that recently emerged in China and South East Asian countries [12, 28]. However, few studies have focused on the evolutionary trends and characterization of PRRSV presenting in Vietnam. Thus, the aim of this study was to investigate the genetic diversity, selective pressure, and glycosylation patterns in GP5 of PRRSV strains that appeared in Vietnam during 2007–2015.

## Methods

### Sample collection

For this study, we used 40 PRRS-positive sera or tissue samples, as confirmed by RT-PCR; the samples were collected from pigs in provinces in North Vietnam during 2011–2015. Total PRRSV RNA was extracted using TRIzol Reagent (Invitrogen, USA) according to the manufacturer’s instruction. Reverse transcription was performed using SuperScript™ III First-Strand Synthesis SuperMix (Thermo Fisher, USA). ORF5 sequences were amplified by RT-PCR using previously described primers [12]. PCR products were directly sequenced (Macrogen, Seoul, Korea). The raw sequences were assembled and aligned using BioEditv7.2.5 [14] against the corresponding ORF5 sequences from GenBank to construct the complete ORF5 sequence. Additional 104 Vietnamese ORF5 reference sequences from field isolates collected from GenBank were also used in this study (Additional file 1: Table S1).

### Phylogenetic analysis and classification

In order to identify the lineage classifications for all the PRRSV strains circulating in Vietnam, an ORF5-based phylogeny was reconstructed using a restricted parameter substitution model [35] with IQ-TREE software [27]. The total data set in this study contained 144 Vietnamese ORF5 gene sequences and 612 worldwide ORF5 reference sequences for lineages 1 to 9 [35]. Bootstrap values were obtained using the ultrafast bootstrap approximation method with 1000 replicates [25] (both programs are available at http://iqtree.cibiv.univie.ac.at/).

### Bayesian phylogenetic inference of ORF5 from Vietnamese strains

The coalescent-based Bayesian Markov Chain Monte Carlo (MCMC) method was used to investigate the phylogenetic relationship among Vietnamese PRRSV strains based on ORF5 sequences. The SRD06 codon-based model was used as a nucleotide substitution model [29, 31] and combined with (i) 5 molecular clock models (Strict clock, uncorrelated lognormal relaxed clock, uncorrelated exponential relaxed clock, random clock, and fixed local clock) and (ii) 7 demographic coalescent tree models (constant size, exponential growth, logistic growth, expansion growth, Bayesian skyline, extended Bayesian skyline plot, and Bayesian skygrid). In each analysis, the MCMC chain (50 million generations, sampling every 5000 stages) was performed using BEAST v1.8.2 software [9]. Five independent runs were done to verify the distribution in the MCMC run. The corresponding output log files were combined by LogCombiner before subsequently analyzing via Tracer v1.6 to select the best-fit data models for molecular clock and coalescent tree priors using Akaike’s information criterion (AICM) analysis with 1000 replicates [2]. A Bayesian phylogenetic tree was selected from combined trees files from the above chosen best-fit models using TreeAnnotator in BEAST package.

### Glycosylation site prediction

Glycosylation sites in the Vietnamese PRRSV strains were predicted using the NetNGlyc server web utility (http://www.cbs.dtu.dk/services/NetNGlyc/). Adefault threshold of 0.5 was used to identify potential N-glycosylation sites, followed by additional thresholds of 0.75 and 0.9 to identify the potential N-glycosylation sites with higher confidence levels.

### Selective pressure

GP5 sites undergoing positive selection were inferred using 5 algorithms: SLAC, FEL, IFEL, FUBAR, and MEME (available on the Datamonkey web server: www.datamonkey.org). Sites undergoing negative selection were predicted using 4 algorithms: SLAC, FEL, IFEL, and FUBAR. To identify other sites undergoing potential selective pressure, sites were analyzed for either diversifying or purifying selection at *P*-value ≤0.1 using SLAC, FEL, IFEL, and MEME methods, or for posterior probability ≥ 90% using the FUBAR method.

## Results

To investigate the evolution of Vietnamese PRRSV strains, we analyzed the time scale phylogenetic tree, the genetic diversity among strains, the time of most common ancestor of PRRSV strains in Vietnam as well as the change of N-glycosylation pattern during this time.

### Phylogenetic analysis of the ORF5 sequence

**Fig. 1:**
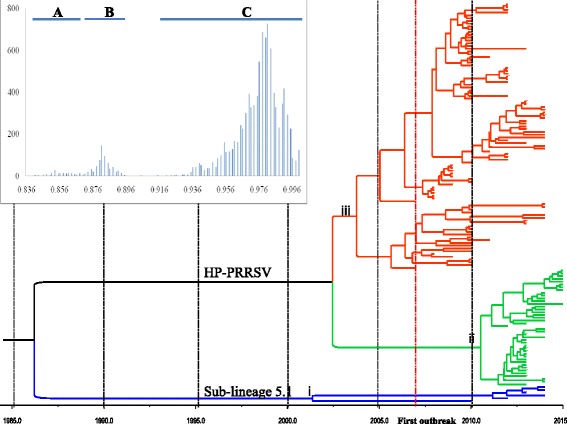
Phylogenetic tree based on nucleotide sequence of the ORF5 gene of 144 PRRSV strains isolated in Vietnam during 2007–2015. The phylogenetic tree, generated via the MCMC method using BEAST v1.8.2 software, identified three different groups. The inserted histogram illustrates pairwise sequence comparisons of Vietnamese PRRSV type 2 strains. Three distinct nucleotide identity distribution peaks are shown. The time-scale (in years) represented in the tree is indicated by the scale bar

Based on the constructed phylogenetic tree, the major PRRSV strains (*n* = 138) isolated in Vietnam could be classified into sub-lineage 8.7, which is closely related to the highly pathogenic PRRSV strains recently isolated in China, including JXA1 and SX2009 [47]. The remaining strains (*n* = 6) were classified into sub-lineage 5.1, which contains VR-2332-related strains [35] (Additional file 2: Figure S1). Further analysis based on Bayesian inference showed that HP-PRRSVs in Vietnam can be divided into two main sub-groups (Fig. 1). Interestingly, most of the PRRSV strains collected in North Vietnam during 2013–2015 belonged to sub-group ii (Fig. 1). Under the best-fit model selected, the substitution rate in the ORF5 gene of the Vietnamese PRRSV strains was about4.459 × 10^−3^ (95% highest posterior density (HPD) intervals: 3.0981 × 10^−3^–5.8523 × 10^−3^). In addition, the geometric mean time to the most recent common ancestor (T_MRCA_) of the HP-PRRSV isolated in Vietnam was approximately 13 years ago and the T_MRCA_ of sub-lineage 5.1 PRRSV strains was more than 16 years ago (95% HPD was 9.2708–18.8409 and 6.9872–32.5917 for the HP-PRRSV group and sub-lineage 5.1 group, respectively).

### Genetic diversity of the Vietnamese PRRSV strains during 2007–2015

Genetic comparison of the ORF5 gene of the Vietnamese PRRSV strains collected from 2007 to 2015 showed that 144Vietnamese PRRSV strains in this study shared 81–100% nucleotide identity (Table 1). Furthermore, the similarity among the ORF5 sequences presented in the same year was about 84.5–100%. Especially in 2010, 2012, 2013, and 2014, when the appearance of PRRSV sub-lineage 5.1 strains was recorded, differences among nucleotide sequences was up to 15.5% while in the remaining years, it was just about 2%. Further analysis showed that the similarity among Vietnamese HP-PRRSV strains was of 91.6–100% while the difference among sub-lineage 5.1 strains was up to 9.5%.

**Table 1:** Nucleotide and deduced amino acid identities among 144 Vietnamese PRRSV strains

Year		2015	2014	2013	2012	2011	2010	2009	2008	2007
2015	nt	99.3–100	88.1–99.6	88.1–99.5	89.1–99.1	98.1–99.1	86–99.1	98.1–99.1	98.1–99.1	98.5–99.1
	aa	98–100	85.5–95.5	85.5–98.5	88–98.5	96.5–99	84–99	97–99	97–99	97.5–98.5
2014	nt		84.8–100	85.1–100	86–99.5	87.8–99.6	84.1–99.1	87.8–99.1	87.8–99.1	88–99.1
	aa		82–100	82–100	84–99.5	85.5–100	81–99.5	86–99.5	86–99.5	85.5–99
2013	nt			86.5–100	87–99.5	87.8–99.3	83.6–99	87.8–99	87.8–99	88–99
	aa			83.5–100	84.5–99.5	86–99	81.5–99.5	86–99	86–99	85.5–98.5
2012	nt				87.8–100	88.8–99.8	84.6–100	88.8–99.5	88.8–99.5	89–98.6
	aa				87–100	88.5–99.5	81–100	88.5–99.5	88.5–99.5	88–99
2011	nt					98.1–100	85.6–100	98.1–99.5	98.1–99.5	98.1–99.3
	aa					98–100	83–100	98–100	98–100	97.5–99.5
2010	nt						84.5–100	85.3–100	85.3–100	85.5–99.3
	aa						81.5–100	83–100	83–100	83.5–99.5
2009	nt							98.1–100	98.1–100	98.1–99.3
	aa							98.5–100	98.5–100	98–99.5
2008	nt								98.1–100	98.1–99.3
	aa								98.5–100	98–99.5
2007	nt									98.5–100
	aa									98.5–100

The deduced amino acid sequence encoded by the ORF5 gene of 144 Vietnamese PRRSV strains shared 82–100% identity. For each sub-lineage group, the amino acid identity was 90–100% and 86.5–100% for HP-PRRSV and sub-lineage 5.1, respectively (Table 1).

### Glycosylation site variants

A total of 7 potential N-glycosylation sites (amino acids 30, 32, 33, 34, 35, 44 and 51) were found for the Vietnamese PRRSV strains isolated in Vietnam during the 2007–2015 period. The identified positions and the total numbers of N-glycosylation sites were diverse. Notably, PRRSV strains isolated during the outbreaks in 2014 had the greatest variation in N-glycosylation patterns, followed by those from the outbreaks in 2013, which had 8 and 7 glycotypes (Table 2). Glycosylation site variations were located between amino acids 32 and 35, while N44 and 51 seemed to be conserved in most of the Vietnamese strains, presenting in 97.2% and 100% of strains, respectively. Furthermore, N41 was predicted as a glycosylation site with higher potential (≥0.75). An N-glycosylation pattern of N30, N35, N44, and N51 seems to be the main glycotype in Vietnamese PRRSV strains, accounting for nearly 61%. Interestingly, we observed differences in the frequencies of N-glycosylation positions between sub-lineage 5.1 strains and sub-lineage 8.7 strains. To be specific, N30, N32, N33, N34, and N35 were identified as potential N-glycosylation sites in sub-lineage 8.7, accounting for 92.09, 2.88, 12.95, 18.71 and 82.73% of strains, respectively, whereas only N30, N33, and N34 were predicted as in sub-lineage 5.1 accounting for 33.33, 66.67, and 33.33% of strains, respectively (data not shown). Furthermore, only two Vietnamese PRRSV sub-lineage 5.1 strains had similar N-glycosylation patterns as the vaccine strain VR2332, while the other strains lacked the potential N-glycosylation site at N30. On the other hand, 88 Vietnamese HP-PRRSV strains had the same N-glycosylation pattern as the JXA1 vaccine strains.

**Table 2:** Glycosylation pattern of PRRSV strains in Vietnam during 2007–2015

Year/Ref Strain	N-glycosylation site							Number of sequence	% of total
	30	32	33	34	35	44	51		
VR-2332	x		x			xxx	x		
JXA1	x				x	xxx	x		
2007	x				x	xx	x	1	0.69 %
	x					xx	x	1	0.69 %
2008	x				x	xx	x	6	4.17 %
2009	x				x	xx	x	7	4.86 %
2010	x				x	xx	x	31	21.53 %
	x			x	x	xx	x	1	0.69 %
	x				x		x	1	0.69 %
				x		xx	x	4	2.78 %
	x		x			xx	x	3	2.08 %
2011	x		x			xx	x	1	0.69 %
	x				x	xx	x	2	1.39 %
2012	x				x	xx	x	11	7.64 %
	x		x			xx	x	2	1.39 %
		x	x			xx	x	3	2.08 %
	x				x		x	1	0.69 %
2013	x			x	x	xx	x	14	9.72 %
	x		x	x		xx	x	2	1.39 %
	x		x			xx	x	1	0.69 %
	x					xx	x	2	1.39 %
	x				x	xx	x	6	4.17 %
			x			xx	x	1	0.69 %
					x	xx	x	3	2.08 %
2014	x				x		x	1	0.69 %
	x		x				x	1	0.69 %
	x			x	x	xx	x	6	4.17 %
	x				x	xx	x	15	10.42 %
	x		x			xx	x	5	3.47 %
		x	x			xx	x	1	0.69 %
			x			xx	x	2	1.39 %
				x		xx	x	1	0.69 %
2015	x				x	xx	x	9	6.25 %

x: indicating the potential N-glycosylation site at cut off value; xx and xxx: indicating the potential N-glycosylation site at additional value (>0.75 and >0.9, respectively)

### Selective pressure in GP5

**Fig. 2:**
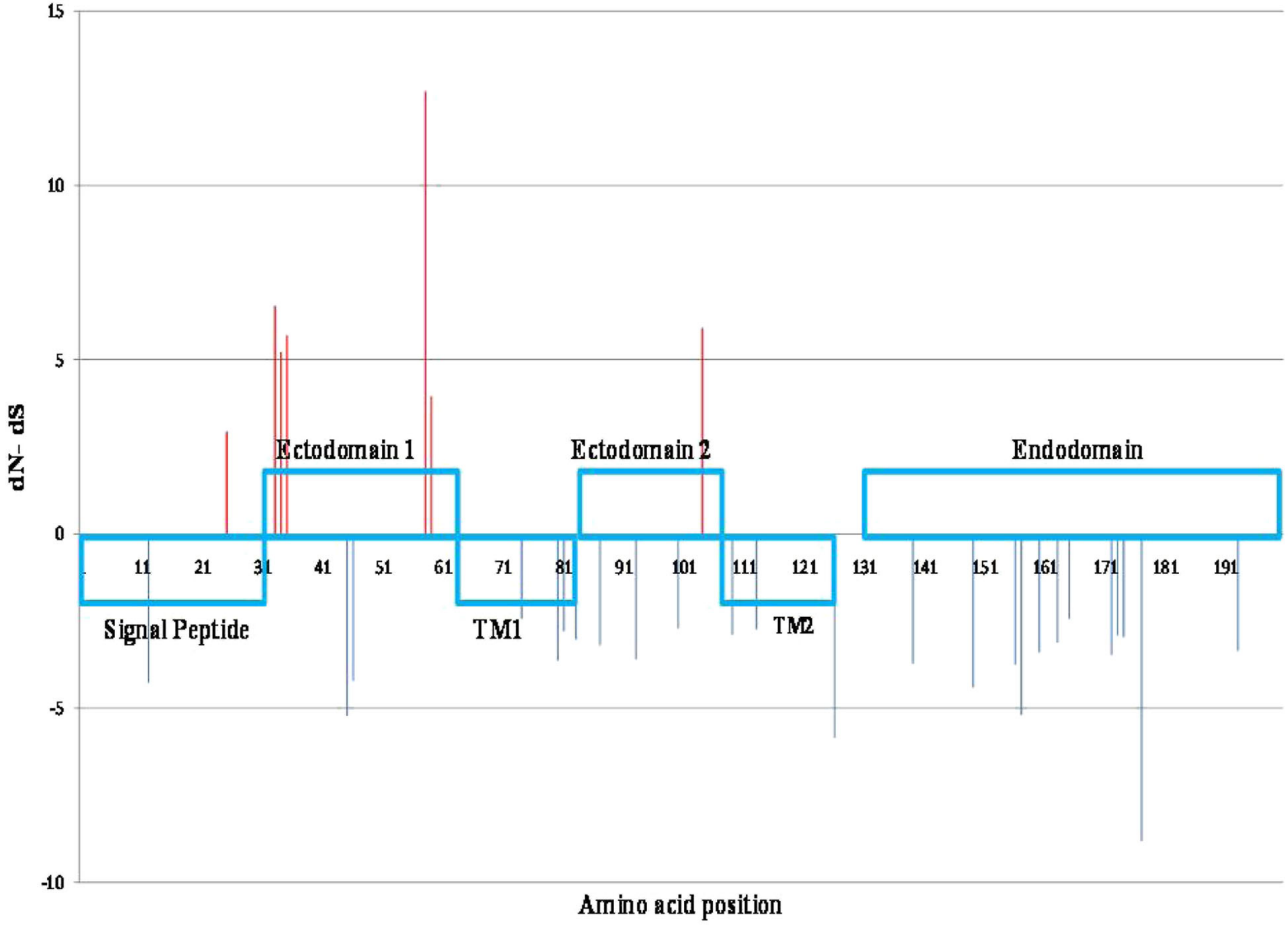
Amino acids under selective pressure. *Upper rectangles* indicate the ecto-domain 1, ecto-domain 2, and endo-domain, whereas *lower rectangles* indicate the signal peptide, trans-membrane 1, and trans-membrane 2 regions. The *red* lines and *blue* lines indicate sites under significant positive and negative selection, respectively. dN-dS represents the normalized dN–dS value according to the FEL method

To identify positions under selective pressure, SLAC, FEL, IFEL, MEME, and FUBAR methods were implemented separately. Since each method utilizes a different algorithm for predicting sites under positive or negative selection, for our study, we considered sites to be undergoing diversifying selection if so predicted by all 5 of the methods, and to be undergoing purifying selection if predicted by 4 of the methods. Consequently, we identified 7 positions as potentially undergoing positive selection (codons 25, 33, 34, 35, 58, 59, and 104). Most of the positive selection sites were located in ecto-domain 1 (*n* = 5), while only 1 site undergoing diversifying selection was found in each ecto-domain 2 and signal domain (Fig. 2).

A different pattern was observed for the negative selection sites. In our study, 25 sites were predicted to be undergoing negative selection (codons 12, 45, 46, 74, 80, 81, 83, 87, 93, 100, 109, 113, 126, 139, 149, 156, 157, 160, 163, 165, 172, 173, 174, 177, and 193). Purifying selection sites were mainly located in the endo-domain (*n* = 12). Furthermore, negative selection sites were detected in trans-membrane 1, trans-membrane 2, and ecto-domain 2 (positions 4, 3, and 3, respectively) (Fig. 2).

## Discussion

Since first identified in the late 1980s, PRRSV has become the most significant porcine reproductive pathogen. The ORF5 gene is the most diverse gene not only in PRRSV, but also in other arteriviruses, and has been an important target for investigations on the genetic characterization and evolution of PRRSV worldwide [34].

In this study, most of the PRRSV strains isolated in Vietnam during 2007–2015 belonged to sub-lineage 8.1, except for 6 strains that belonged to sub-lineage 5.1 (Additional file 2: Figure S1). This result is consistent with that of previous reports, indicating that most of the PRRSV strains isolated in Vietnam are close related to JXA1 [12, 28]. In fact, attenuated vaccine strains belonging to sub-lineage 5.1, such as VR2332 and BSL-PS, have been approved for use in Vietnam. Furthermore, although HP-PRRSV strains are the main agents of PPRS in countries around Vietnam, such as China and others in South East Asia, strains from other type 2 PRRSV lineages such as lineages 1, 3, and 5 have also circulated [22, 40]. Therefore, these data suggest that the appearance of sub-lineage 5.1 in Vietnam may be due to vaccine descendants or commercial activities. In addition, our study indicates that the HP-PRRSV strains circulating during 2013–2015 were distantly related to the other HP-PRRSV strains. This may have resulted from the introduction of new PRRSV strains into Vietnam. However, the limited number of Vietnamese PRRSV ORF5 sequences used in this study may not exactly reflect the genetic diversity of PRRSVs in Vietnam.

Although, the PRRSV strains circulating in Vietnam clustered within sub-lineage 5.1 and 8.7, their percentages of intra-sub-lineage genetic diversity were 90.5–100% and 91.6–100%, respectively. The intra-sub-lineage genetic diversity in our study was higher than in a previous study [35]. This result might be due to the high substitution rate detected in the ORF5 gene. In addition, the substitution rate in the ORF5 gene of the Vietnamese strains, which was 4459 × 10^−3^, was slightly faster than the substitution rates observed in common type II PRRSV strains [31] This supports our hypothesis. According to our analysis, the T_MRCA_ of sub-lineage 5.1 strains was approximately 17 years ago, which is supported by serological evidence for anti-PRRSV antibodies in Vietnam during this time. The T_MRCA_ of sub-lineage 8.7 strains was estimated to have occurred in 2002, which is similar to the T_MRCA_ of the HP-PRRSV from China [36].

It is reported that the N-glycosylation positions in GP5 affect the adaptation of PRRSV to the host’s immune response and infectivity [1]. In our study, 11 potential N-glycotypes were observed for the GP5 protein of the Vietnamese PRRSV strains isolated between 2007 and 2015 (Table 2). Furthermore, our investigation revealed diversity in the putative N-glycosylation site amino acid positions (7 different positions) and quantity (3 to 5 sites). However, the N-glycotype diversity identified in the Vietnamese PRRSV strains was less than that recorded for the PRRSV strains from Eastern Canada isolated between 1998 and 2009 [6]. Another report shows that the PRRSV strains isolated in China from 2006 to 2009 have the same N-glycosylation sites as the Vietnamese strains [26]. In all positions, the N44 site was predicted at a high confidence level (≥0.75) and seemed to be conserved. These results are consistent with the important role of this residue in infectivity [1]. The N51 glycosylation site has also been demonstrated to affect the growth kinetics of PRRSV [1], and is highly conserved in type 2 PRRSV from many countries [6, 17, 19].

On the other hand, N-glycosylation sites seem to vary at positions 32–35 (Table 2). These sites are located within the hyper-variable region that has been previously described [39]. It is believed that the variations in N-glycosylation in this region may influence viral neutralization [16]. However, not all potential glycosylation sites in this region are glycosylated. Li et al. [20] suggested that only 2 or 3 glycosylation sites in this region are utilized and that their exact positions are still unknown.

Another notable result of this study was the distribution of diversity and purified selection positions throughout the GP5 of Vietnamese PRRSV strains. Nguyen et al. [31] can not conclude the role of selected position in typical PRRSV and HP-PRRSV. In this study, our analysis of sites under selective pressure indicated that most of the sites undergoing positive selection (amino acids 33, 34, 35, 58, and 59) are located in ecto-domain 1, which contains the linear epitope, and an additional positive selection site is also predicted in ecto-domain 2 (amino acid 104). Our results are generally consistent with those of previous studies, with the exception of the positive selection site predicted in the signal peptide [6, 15, 26]. A previous study demonstrated that site-directed mutagenesis of the amino acid residues at 102 and 104 can enhance PRRSV evasion of neutral antibodies in vitro [10]. This supports the results from our current study. In addition, the potential N-glycosylation sites at the N33, N34, and N35 have been previously identified as undergoing positive selection in the HP-PRRSV strains recently isolated in China [26, 45]. In a previous investigation, in vitro neutralization experiments showed that mutation of the N34 Asp (wt) to Asn slightly decreases the neutralizing activity of Asp-34 sera [33].

Our study showed that the main locations under negative selection were in the endo-domain, following by trans-membrane 1, trans-membrane 2, and ecto-domain 2. This agrees with the findings of Xu et al. [45]. Negative selective pressure within the trans-membrane domains may relate to the integrity or functionality of the virion whereas the distribution of sites under purifying selection in the endo-domain could relate to the budding process of PRRSV. A similar function has been observed in alphaviruses, where E2 and the nucleocapsid protein specifically interact with each other [37]. In addition, mutations within endo-domain of glycoprotein E2 affected the biological characteristic of Sindbis virus [21, 43].

## Conclusions

This study first describes the molecular evolution of ORF5 of PRRSV occurred in Vietnam since the first outbreaks. Phylogenetic analysis based on ORF5 sequence indicated the diversity of PRRSV in Vietnam. Furthermore, the variance of N-glycosylation sites and position under selective pressure were demonstrated. This study expands existing knowledge on the genetic diversity and evolution of PRRSV in Vietnam and assists the effective strategies for PRRS vaccine development in Vietnam.

## Additional files

Additional file 1: Table S1. The information of Vietnamese PRRSV strains using in this study. (PDF 99 kb)
Additional file 2: Figure S1. Classification of Vietnamese PRRSV strains based on the reference sequences. (PDF 158 kb)

## Abbreviations

HP: Highly pathogenic
HPD: Highest posterior density
MCMC: Markov Chain Monte Carlo
ORF: Open reading frame
PRRS: Porcine reproductive and respiratory syndrome
PRRSV: Porcine reproductive and respiratory syndrome virus
T_MRCA_: Time to the most recent common ancestor
