# Supplementary material for: Molecular evolution of type 2 porcine reproductive and respiratory syndrome viruses circulating in Vietnam from 2007 to 2015
Source: BMC Vet Res. 2016 Nov 17;12:256. doi: 10.1186/s12917-016-0885-3 (PMC5112882; doi:10.1186/s12917-016-0885-3)
Supplement: Additional file 1: Table S1. — The information of Vietnamese PRRSV strains using in this study. (PDF 99 kb) [file 12917_2016_885_MOESM1_ESM.pdf]

Molecular Evolution of Type 2 Porcine Reproductive and Respiratory Syndrome VirusesCirculating in Vietnam from 2007 to 2015

*Hai Quynh Do[1], Dinh Thau Trinh[1], Thi Lan Nguyen[1], Thi Thu Hang Vu[2], Duc Duong Than[2], Thi Van Lo[2], Minjoo Yeom[3], Daesub Song[3], SeEun Choe[4], Dong-Jun An[4], Van Phan Le[1]**

Journal: BMC Veterinary Research

*Corresponding Author: Dr. Van Phan Le
Faculty of Veterinary Medicine, Vietnam National University of Agriculture (VNUA), Vietnam
Telephone: +84-914-938-793; Fax: +84-43.67625883; E-mail: letranphan@vnua.edu.vn

Supplement table 1: Vietnamese PRRSV strain used in this study

No	Strain	Location (Province)	Year	Acession number	Note
1	HUA/HP23	Hung Yen	2013	KX424897	This study
2	HUA/HP22	Hung Yen	2013	KX424898	This study
3	HUA/HP21	Hung Yen	2013	KX424899	This study
4	HUA/HP20	Hung Yen	2013	KX424900	This study
5	HUA/HP19	Hung Yen	2013	KX424901	This study
6	HUA/HP38	Thai Binh	2015	KX424882	This study
7	MUA/HP26	Son La	2014	KX424894	This study
8	HUA/HP29	Son La	2014	KX424891	This study
9	HUA/HP28	Son La	2014	KX424892	This study
10	HUA/HP27	Son La	2014	KX424893	This study
11	HUA/HP6	Hai Duong	2013	KX424902	This study
12	HUA/HP7	Hai Duong	2013	KX424903	This study
13	HUA/HP9	Hai Phong	2013	KX424904	This study
14	HUA/HP11	Hai Phong	2013	KX424906	This study
15	HUA/HP14	Hai Duong	2012	KX424912	This study
16	HUA/HP13	Yen Bai	2012	KX424915	This study
17	HUA/HP16	Dien Bien	2012	KX424913	This study
18	HUA/HP5	Lao Cai	2012	KX424914	This study
19	HUA/HP8	Nghe An	2013	KX424908	This study
20	HUA/HP10	Hai Phong	2013	KX424905	This study
21	HUA/HP12	Hanoi	2013	KX424907	This study
22	HUA/HP15	Hanoi	2013	KX424909	This study
23	HUA/HP18	Hanoi	2013	KX424910	This study
24	HUA/HP17	Hanoi	2013	KX424911	This study
25	HUA/HP24	Hai Duong	2014	KX424896	This study
26	HUA/HP37	Bac Ninh	2015	KX424883	This study
27	HUA/HP36	Bac Ninh	2015	KX424884	This study
28	HUA/HP35	Bac Ninh	2015	KX424885	This study
29	HUA/HP34	Bac Ninh	2015	KX424886	This study
30	HUA/HP33	Bac Ninh	2015	KX424887	This study
31	HUA/HP32	Bac Ninh	2015	KX424888	This study
32	HUA/HP31	Bac Ninh	2015	KX424889	This study
33	HUA/HP30	Bac Ninh	2015	KX424890	This study
34	HUA/HP25	Hai Duong	2014	KX424895	This study
35	HUA/HP1	Bac Giang	2013	KF699846	This study

36	HUA/HP1963	Lao Cai	2011	KF699844	This study
37	HUA/HP2	Bac Giang	2013	KF699847	This study
38	HUA/HP2228	Hanoi	2012	KF699845	This study
39	HUA/HP3	Vinhphuc	2013	KF699848	This study
40	HUA/HP4	Vinhphuc	2013	KF699849	This study
41	07QN	Quang Nam	2007	FJ394029	Feng *et al.*, 2008
42	10Bali	Bac Lieu	2010	KF523295	
43	10HuY	Hung Yen	2010	KF523293	
44	10NgAn	Nghe An	2010	KF523296	
45	10QuNi	Quang Ninh	2010	KF523297	
46	10TiGi	Tien Giang	2010	KF523294	
47	171NA	Nd[‡]	2010	AB588638	
48	347KS	Nd	2010	AB588637	
49	347TKS	Nd	2010	AB588636	
50	4793TG	Ho Chi Minh City	2010	HQ700874	
51	5076HCM	Ho Chi Minh City	2010	HQ700875	
52	5077HCM	Ho Chi Minh City	2010	HQ700876	
53	5111HCM	Ho Chi Minh City	2010	HQ700877	
54	5112HCM	Ho Chi Minh City	2010	HQ700878	
55	5172HCM	Ho Chi Minh City	2010	HQ700879	
56	5215HCM	Ho Chi Minh City	2010	HQ700880	
57	5251HCM	Ho Chi Minh City	2010	HQ700881	
58	5280DAKN	Ho Chi Minh City	2010	HQ700882	
59	5321HCM	Ho Chi Minh City	2010	HQ700883	
60	5339HCM	Ho Chi Minh City	2010	HQ700884	
61	5386BT	Ho Chi Minh City	2010	HQ700885	
62	5402BD	Ho Chi Minh City	2010	HQ700886	
63	5445HCM	Ho Chi Minh City	2010	HQ700887	
64	AGES/568-30FC/13	Nd	2013	KM588915	
65	BDR1	Binh Duong	2010	JQ860381	Nguyen *et al.*, 2013
66	CTC1	Can Tho	2012	JQ860382	Nguyen *et al.*, 2013
67	CTC2	Can Tho	2012	JQ860383	Nguyen *et al.*, 2013
68	CTHS1	Can Tho	2012	JQ860384	Nguyen *et al.*, 2013
69	CTHS2	Can Tho	2012	JQ860385	Nguyen *et al.*, 2013
70	CTHS3	Can Tho	2012	JQ860386	Nguyen *et al.*, 2013
71	D1/HCM1/VN2013	Ho Chi Minh City	2013	KR261789	Do *et al.*, 2015
72	D10/BD1/VN	Binh Duong	2013	KR261796	Do *et al.*, 2015
73	D11/HT2/VN2013	Hanoi	2013	KR261784	Do *et al.*, 2015
74	D12/HY/VN2013	Hung Yen	2013	KR261804	Do *et al.*, 2015
75	D13/BD2/VN	Binh Duong	2014	KR261795	Do *et al.*, 2015
76	D14/DN12/VN	Dong Nai	2014	KR261780	Do *et al.*, 2015
77	D15/BD3/VN	Binh Duong	2014	KR261790	Do *et al.*, 2015
78	D16/HCM2/VN	Ho Chi Minh City	2014	KR261794	Do *et al.*, 2015
79	D18/ST0/VN	Soc Trang	2014	KR261803	Do *et al.*, 2015
80	D20/BDi1/VN	Binh Dinh	2014	KR261785	Do *et al.*, 2015
81	D21/BT1/VN	Binh Thuan	2014	KR261781	Do *et al.*, 2015
82	D22/ST1/VN	Soc Trang	2014	KR261800	Do *et al.*, 2015
83	D23/Btr1/VN	Ben Tre	2014	KR261791	Do *et al.*, 2015
84	D24/BD4/VN	Binh Duong	2014	KR261798	Do *et al.*, 2015

85	D27/BT2/VN	Binh Thuan	2014	KR261799	Do *et al.*, 2015
86	D28/BT3/VN	Binh Thuan	2014	KR261775	Do *et al.*, 2015
87	D29/DN5/VN	Dong Nai	2014	KR261772	Do *et al.*, 2015
88	D3/BRTV1/VN2013	Ba Ria Vung Tau	2013	KR261797	Do *et al.*, 2015
89	D30/BD5/VN	Binh Duong	2014	KR261793	Do *et al.*, 2015
90	D31/BD6/VN	Binh Duong	2014	KR261805	Do *et al.*, 2015
91	D32/ST2/VN	Soc Trang	2014	KR261786	Do *et al.*, 2015
92	D33/HCM3/VN	Ho Chi Minh City	2014	KR261773	Do *et al.*, 2015
93	D41/BT4/VN	Binh Thuan	2014	KR261782	Do *et al.*, 2015
94	D42/DN6/VN	Dong Nai	2014	KR261774	Do *et al.*, 2015
95	D43/DN7/VN	Dong Nai	2014	KR261792	Do *et al.*, 2015
96	D44/DN8/VN	Dong Nai	2014	KR261787	Do *et al.*, 2015
97	D49/DN9/VN	Dong Nai	2014	KR261779	Do *et al.*, 2015
98	D50/BD7/VN	Binh Duong	2014	KR261783	Do *et al.*, 2015
99	D51/DN10/VN	Dong Nai	2014	KR261802	Do *et al.*, 2015
100	D54/DN11/VN	Dong Nai	2014	KR261777	Do *et al.*, 2015
101	D8/DN3/VN	Dong Nai	2014	KR261776	Do *et al.*, 2015
102	D9/AG1/VN2013	An Giang	2013	KR261788	Do *et al.*, 2015
103	D9/AG2/VN2013	An Giang	2013	KR261801	Do *et al.*, 2015
104	DN1	Dong Nai	2010	JQ860375	Nguyen *et al.*, 2013
105	DN11	Dong Nai	2010	JQ860378	Nguyen *et al.*, 2013
106	DN1107	Dong Nai	2009	JQ860373	Nguyen *et al.*, 2013
107	DN1155	Dong Nai	2009	JQ860374	Nguyen *et al.*, 2013
108	DN153	Dong Nai	2008	JQ860371	Nguyen *et al.*, 2013
109	DN292	Dong Nai	2009	JQ860372	Nguyen *et al.*, 2013
110	DN4	Dong Nai	2010	JQ860376	Nguyen *et al.*, 2013
111	DN42	Dong Nai	2009	JQ860367	Nguyen *et al.*, 2013
112	DN44	Dong Nai	2009	JQ860368	Nguyen *et al.*, 2013
113	DN444	Dong Nai	2008	JQ860362	Nguyen *et al.*, 2013
114	DN452	Dong Nai	2008	JQ860363	Nguyen *et al.*, 2013
115	DN460	Dong Nai	2008	JQ860364	Nguyen *et al.*, 2013
116	DN499	Dong Nai	2008	JQ860365	Nguyen *et al.*, 2013
117	DN52	Dong Nai	2010	JQ860377	Nguyen *et al.*, 2013
118	DN59	Dong Nai	2009	JQ860369	Nguyen *et al.*, 2013
119	DN649	Dong Nai	2008	JQ860366	Nguyen *et al.*, 2013
120	DN88	Dong Nai	2009	JQ860370	Nguyen *et al.*, 2013
121	DT7	Dong Thap	2012	JQ860387	Nguyen *et al.*, 2013
122	DT8	Dong Thap	2012	JQ860388	Nguyen *et al.*, 2013
123	DT9	Dong Thap	2012	JQ860389	Nguyen *et al.*, 2013
124	HCM3416	Binh Duong	2010	HQ540650	
125	HCMC2394	Ho Chi Minh City	2010	HQ540653	
126	HCMC3336	An Giang	2010	HQ540646	
127	HCMC3341	Ho Chi Minh City	2010	HQ540647	
128	HCMC3374	Ho Chi Minh City	2010	HQ540648	
129	HCMC3411	Binh Duong	2010	HQ540649	
130	HCMC3440	Binh Duong	2010	HQ540651	
131	HCMC3703	Binh Duong	2010	HQ540652	
132	HCMCC3	Ho Chi Minh City	2010	JQ860379	Nguyen *et al.*, 2013
133	HCMD06	Ho Chi Minh City	2010	JQ860380	Nguyen *et al.*, 2013

134	HGRV1	Hau Giang	2012	JQ860390	Nguyen *et al.*, 2013
135	HGRV2	Hau Giang	2012	JQ860391	Nguyen *et al.*, 2013
136	HUAVetlabPRRS1	Nd	2011	AB856283	
137	HUAVetlabPRRS2	Nd	2011	AB856284	
138	HUAVetlabPRRS3	Nd	2012	AB856285	
139	HUAVetlabPRRS4	Nd	2012	AB856286	
140	MB6	Nd	2010	KM244761	
141	MN1	Nd	2013	KM244763	
142	NA/VIE/V001/2010	Nd	2010	KF698650	
143	NA/VIE/V056/2010	Nd	2010	KF698651	
144	SRV07	Nd	2007	JX512910	

‡: Not determined
