# Supplementary material for: Molecular evolution of type 2 porcine reproductive and respiratory syndrome viruses circulating in Vietnam from 2007 to 2015
Source: BMC Vet Res. 2016 Nov 17;12:256. doi: 10.1186/s12917-016-0885-3 (PMC5112882; doi:10.1186/s12917-016-0885-3)
Supplement: Additional file 2: Figure S1. — Classification of Vietnamese PRRSV strains based on the reference sequences. (PDF 158 kb) [file 12917_2016_885_MOESM2_ESM.pdf]

Molecular Evolution of Type 2 Porcine Reproductive and Respiratory Syndrome Viruses Circulating in Vietnam from 2007 to 2015

*Hai Quynh Do[1], Dinh Thau Trinh[1], Thi Lan Nguyen[1], Thi Thu Hang Vu[2], Duc Duong Than[2], Thi Van Lo[2], Minjoo Yeom[3], Daesub Song[3], SeEun Choe [4], Dong-Jun An[4], Van Phan Le[1]**

Journal: BMC Veterinary Research

* Corresponding Author: Dr. Van Phan Le
Faculty of Veterinary Medicine, Vietnam National University of Agriculture (VNUA), Vietnam
Telephone: +84-914-938-793; Fax: +84-43.67625883; E-mail: letranphan@vnua.edu.vn

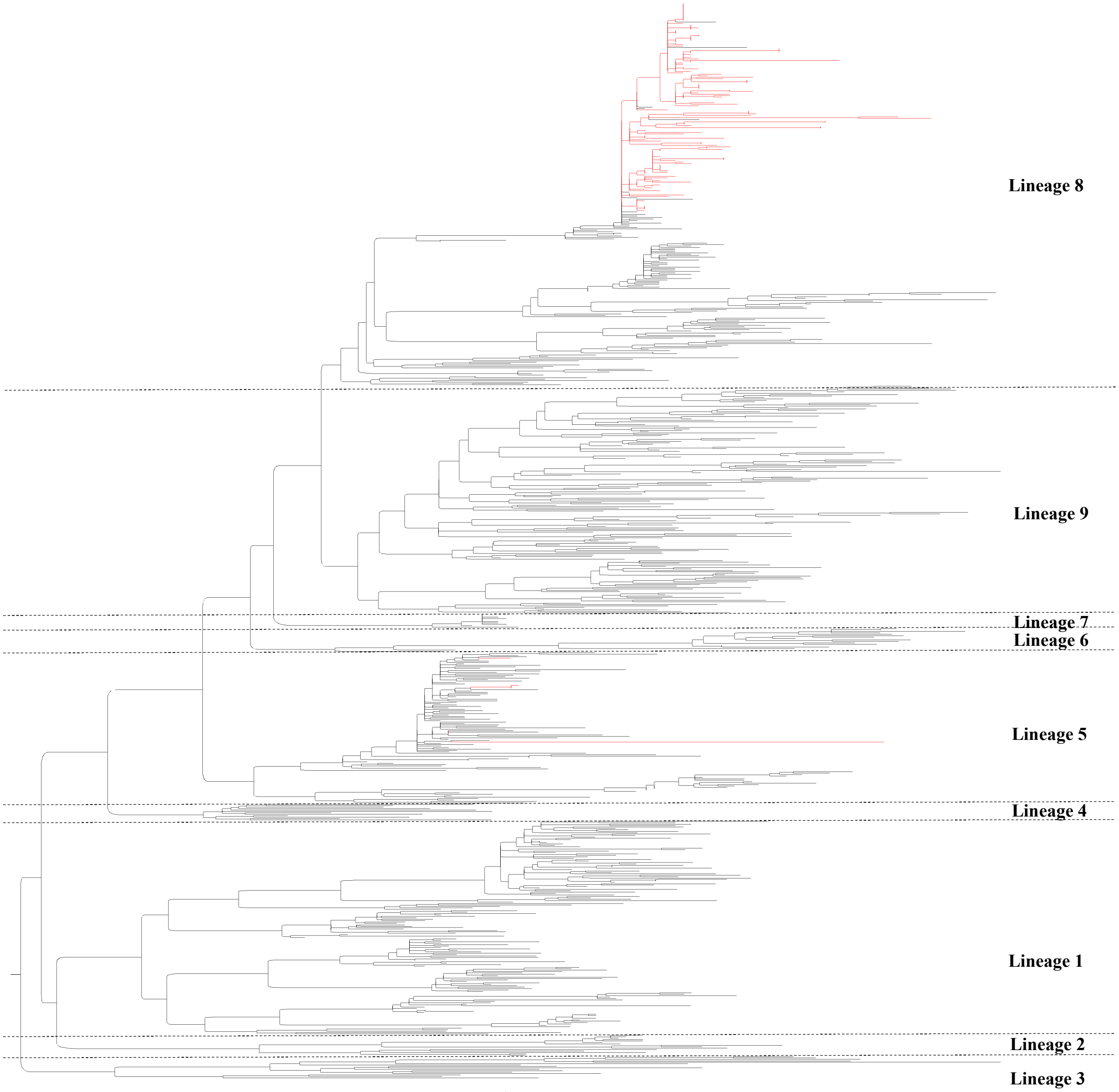

Supplement figure 1: Classification of Vietnamese PRRSV strains based on the reference sequence (Shi *et al.*, 2010b). The red line indicated Vietnamese PRRSV strains. Our result showed that Vietnamese strains clearly belonged to lineage 5 and lineage 8
